# Propolis Envelope Promotes Beneficial Bacteria in the Honey Bee (*Apis mellifera*) Mouthpart Microbiome

**DOI:** 10.3390/insects11070453

**Published:** 2020-07-18

**Authors:** Hollie Dalenberg, Patrick Maes, Brendon Mott, Kirk E. Anderson, Marla Spivak

**Affiliations:** 1 Department of Entomology, University of Minnesota, St Paul, MN 55108, USA; dalen034@umn.edu (H.D.); spiva001@umn.edu (M.S.); 2 Department of Entomology, University of Arizona, Tucson, AZ 85721, USA; pmaes@email.arizona.edu; 3 USDA-ARS Carl Hayden Bee Research Center, Tucson, AZ 85719, USA; Brendon.Mott@ars.usda.gov

**Keywords:** social immunity, antimicrobial resins, mouthparts, mouthpart bacteria, microbiome, propolis

## Abstract

Honey bees collect and apply plant resins to the interior of their nest cavity, in order to form a layer around the nest cavity called a propolis envelope. Propolis displays antimicrobial activity against honey bee pathogens, but the effect of propolis on the honey bee microbiome is unknown. Honey bees do not intentionally consume propolis, but they do manipulate propolis with their mouthparts. Because honey bee mouthparts are used for collecting and storing nectar and pollen, grooming and trophallaxis between adults, feeding larvae, and cleaning the colony, they are an important interface between the bees’ external and internal environments and serve as a transmission route for core gut bacteria and pathogens alike. We hypothesized that the antimicrobial activity of an experimentally applied propolis envelope would influence the bacterial diversity and abundance of the worker mouthpart microbiome. The results revealed that the mouthparts of worker bees in colonies with a propolis envelope exhibited a significantly lower bacterial diversity and significantly higher bacterial abundance compared to the mouthparts of bees in colonies without a propolis envelope. Based on the taxonomic results, the propolis envelope appeared to reduce pathogenic or opportunistic microbes and promote the proliferation of putatively beneficial microbes on the honey bee mouthparts, thus reinforcing the core microbiome of the mouthpart niche.

## 1. Introduction

Honey bees and other social insect colonies have evolved behavioral, physiological, and organizational mechanisms to reduce pathogen transmission, known as social immunity ([1,2] Anderson et al., 2020 this issue). For *Apis mellifera*, one mechanism is the collection of antimicrobial plant resins [3] produced by some plants to defend against phytopathogens [4]. Foraging bees from *A. mellifera* colonies, especially feral colonies that nest in tree cavities, collect large quantities of antimicrobial plant resins and deposit them as a thin continuous layer coating the rough interior walls of the nest cavity, called a propolis envelope [5]. 

Previous research has established the benefits of a propolis envelope for colony health and disease resistance. Propolis in the colony is correlated or associated with increases in colony strength, vitellogenin levels, the antimicrobial activity of larval food, adult bee longevity, brood survival rates, hygienic behavior, and honey production [6,7,8,9,10,11]. Additionally, a reduction in deformed wing virus titers was found in colonies with propolis (even though *Varroa destructor* mite infestation did not change [12]). After an experimental pathogen challenge, colonies with a propolis envelope had fewer clinical signs of chalkbrood [2] and American foulbrood [7] compared to colonies without a propolis envelope. Simone et al. [3] and Borba et al. [6] found that colonies with a propolis envelope exhibited a reduced investment in individual bee immune function. It was hypothesized (but untested) that propolis reduced the amount of opportunistic and/or pathogenic bacteria in the nest, which subsequently reduced the need for bees to activate the physiologically costly immune system [13]. Recently, microbiome sequencing of bees by Borba et al. [6] revealed that a propolis envelope significantly altered the presence of several key members of the gut microbiome and reduced the microbial diversity, suggesting that propolis may promote a healthy gut microbiome [14]. 

To further test if propolis affects the bacterial community composition within a honey bee colony, we chose to investigate the bacteria associated with the mouthparts. Honey bee mouthparts serve as a horizontal transmission route for opportunistic and/or pathogenic, as well as beneficial, bacteria in the nest [15,16]. Honey bee mouthparts are used for a number of important social interactions and nutritional functions: Feeding hypopharyngeal glandular secretion to developing larvae, grooming and trophallaxis between adults, collecting and storing nectar and pollen, and removing debris from the colony. Additionally, the mouthparts form the interface between the gut microbiome and various hive microbiome niches [15,16]. 

The hindgut alone comprises >99% of the bacteria in adult workers; however, there are other important niches where certain bacterial species are consistently found in and around honey bees, including the upper alimentary tract of the bee (mouthparts, hypopharyngeal glands, and crop) and the hive environment (royal jelly, larvae, fresh and stored pollen, and honey) [15,16,17,18,19,20,21]. The bacteria in the hindgut niches intimately interact with host tissue and are exposed to a refined post-digestion assortment of compounds, whereas bacteria in the hive- and mouthpart-associated niches experience many of these compounds directly, have fewer direct host–tissue interactions, and may indirectly benefit the host by outcompeting pathogens, thereby reducing pathogen transmission. These hive- and mouthpart-associated niches differ from the hindgut niche in many ways in that they typically contain far fewer bacteria, but those bacteria experience increased competition with other microbes and are more exposed to antimicrobial compounds such as oxygen, honey, and hypopharyngeal secretions. Bacteria consistently found in these foregut and hive niches are *Bombella apis* (previously named *Acetobacteraceae* Alpha2.2 and *Parasaccharibacter apium* [22,23,24]), *Lactobacillus kunkeei*, and *Fructobacillus fructosus* [18,19,25,26]. 

The mouthpart microbiome is predictive of dysbiosis occurring throughout the gut of the organism [15]. When the highly predictable worker hindgut microbiome is in a state of dysbiosis, the mouthpart microbiome undergoes significant changes in the community structure. Since the honey bee gut microbiota have several functions (protection from opportunists, host adhesion and colonization, immune system and hormone regulation, and enriched metabolism of complex and toxic sugars [20]), it can be inferred that a change in the mouthpart microbiome may be a reliable indicator of honey bee health [15]. 

A propolis envelope may result in different effects on the different honey bee microbial niches. For instance, the mouthpart microbiome may be more directly affected by the antibacterial effects of propolis compared to the gut microbiome, since the mouthparts directly come into contact with propolis during resin collection and application [27]. 

We hypothesized that the presence of a propolis envelope in field colonies would alter the diversity and abundance of bacteria in the honey bee mouthpart microbiome. More specifically, because the mouthpart microbiome of *A. mellifera* has likely adapted to the antimicrobial properties of propolis in the nest cavity over evolutionary time, we hypothesized that pathogenic and opportunistic bacteria would be reduced on the mouthparts of worker bees exposed to propolis compared to bees without a propolis envelope. 

## 2. Materials and Methods

Experimental Design: Six colonies of honey bees derived from an Italian stock (*Apis mellifera ligustica*) were purchased as packages from a northern California beekeeping operation. The colonies were hived in standard Langstroth bee boxes, in April 2018, at the Bee Lab apiary at the University of Minnesota, Saint Paul, MN, U.S.A. Each colony began with one deep box, new wooden frames, and a new plastic foundation (Mann Lake Ltd., Hackensack, MN, USA). Three colonies were provided with an experimental propolis envelope (treatment = P+) by coating the interior of each box with 100 mL of a 30% propolis extract in 70% ethanol solvent, and by fitting propolis-filled traps (see the section on propolis, below) onto the inner two sides and the back wall of the box. The front wall was not given a propolis-filled trap to facilitate removal of the frames for colony management. Three additional colonies were not provided with propolis envelopes (control colonies = P−), but 100 mL 70% ethanol, for the propolis extract was painted on all four inner walls. Natural propolis deposits between boxes and between frames were not removed from either the P+ or P− colonies. Second boxes with the same treatments were added to both sets of colonies in June of 2018 to accommodate colony growth. These were similar methods to those used in previous propolis envelope experiments [3,6].

Propolis: The propolis used to form the propolis envelope was collected from colonies located at the University of Minnesota Outreach Research and Education Park (UMore) in Rosemount, MN, in the summer of 2017. Commercial propolis traps (Mann Lake Ltd., MN, USA) were placed over the top frames of the colonies and at the end of the season, the propolis-filled traps were removed and stored at −80 °C. Nine of the propolis-filled traps were cut to fit (1.5 traps/P+ box for two deeps) within the three P+ experimental colonies, as described above. The propolis was harvested from the remaining 15 traps to make the propolis ethanol extract. The frozen resin was twisted out of the traps and pulverized using a coffee grinder. A 30% (*w*/*v*) ethanol extract of propolis was made by dissolving 30 g of the powdered propolis in 70 mL of 70% ethanol. Propolis was dissolved in the dark for 3 days and was shaken once manually each day. Debris was removed from the propolis extract using filter paper under vacuum filtration. The final concentration of propolis was 196 mg/mL, determined by allowing triplicate samples of the propolis extract to dry in a laminar flow hood until the weight no longer changed. In vitro antibacterial activity of the propolis ethanol extract was validated using an antibacterial assay against *Paenibacillus larvae* following the methods used in Wilson et al. [28] (Supplementary Material Table S1). 

Bee Mouthpart Samples: In July 2018, frames of an emerging brood were removed from the six experimental colonies and were placed in individual screened cages and allowed to emerge overnight in an incubator at 32 °C and 50% relative humidity. The next day, the newly emerged bees were marked with enamel paint on the thorax and returned to their source colony. Nine days later, the marked bees were collected from the colonies, individually placed in 2 mL Eppendorf tubes, and stored at −80 °C. Frozen bees from the colonies (n = 92) were individually thawed and then decapitated; the bodies were returned to the freezer. The proboscis was extended from the head using sterile tweezers and the esophagus was cut proximal of the proboscis at the cardo with sterile dissection scissors. The mandibles were not included, in order to be comparable to Anderson et al. [16]. Dissected mouthparts were placed in tubes containing 300 μL of sterile 1X TE buffer and 100 μL of 0.1 mm beads and returned to the freezer until homogenization.

DNA extraction: Frozen mouthparts were thawed and put in a beadbeater at 30 s intervals for a total of 2 min. A total of 100 μL lysis buffer (20 mM Tris-HCl, 2 mM EDTA, 5% Triton X-100, 80 mg/mL lysozyme, pH 8.0) was added to each sample and the samples were incubated at 37 °C for 30 min. DNA was then purified using a Thermo Fisher Scientific GeneJet Genomic DNA Purification Kit, according to the manufacturer’s instructions for gram-positive bacteria. DNA was eluted with 100 uL Elution Buffer and stored at −20 °C until quantification and characterization. 

16S rRNA gene sequencing for absolute community abundance: The absolute community abundance of each sample was quantified with a quantitative real-time PCR (qPCR) assay of 16S rRNA gene copies [29]. A 466 bp fragment in the V3–V4 region of the bacterial rRNA gene was amplified from total DNA using the BactQuant primer pair (forward primer, 5′-CCTACGGGDGGCWGCA-3′; reverse primer, 5′-GGACTACHVGGGTMTCTAATC-3′). qPCRs were carried out on a BioRad CFX96 thermocycler in 20 μL reactions containing 6.4 μL of DEPC water, 10 μL of iTaq Universal SYBR Green Supermix (BioRad), 0.8 μL of forward primer, 0.8 μL of reverse primer, and 2 μL of DNA template. The cycling conditions were 95 °C for 3 min, followed by 40 cycles of 95 °C for 10 s and 60 °C for 60 s. A standard curve was generated using a 10-fold serial dilution series of a plasmid standard containing the full-length *Escherichia coli* 16S rRNA gene. The logarithmic regression equation generated from the 10-fold serial dilution plasmid series was used to convert qPCR-generated cycle thresholds into 16S copy numbers. The qPCR results were expressed as the total number of 16S rRNA gene copy numbers per DNA extraction (100 μL volume elution). Any qPCR amplifications with inconsistent melt curves were discarded.

16S rRNA gene sequencing for community analysis: The V3–V4 region of the 16S rRNA gene was amplified using PCR primers (forward primer, 341F 5′-CCTACGGGNGGCWGCAG-3′; reverse primer, 805R 5′-GACTACHVGGGTATCTAATCC-3′). Amplification was performed using the HotStarTaq Plus Master Mix Kit (Qiagen, USA) under the following conditions: 94 °C for 3 min, followed by 28 cycles of 94 °C for 30 s, 53 °C for 40 s, and 72 °C for 1 min, with a final elongation step at 72 °C for 5 min. PCR products were confirmed using a 2% agarose gel. PCR products were used to prepare DNA libraries following Illumina TruSeq DNA library preparation. Sequencing was performed on a MiSeq at the University of Arizona Genetics Core. All sequence data were deposited in GenBank under Sequence Read Archive (SRA) accession PRJNA594720.

16S rRNA gene sequence analysis: Sequences were processed using MOTHUR v.1.39.5 [30]. Forward and reverse reads were joined using the make.contigs command. After the reads were joined, the first and last five nucleotides were removed using the SED command in UNIX. Using the screen.seqs command, sequences were screened to remove ambiguous bases. Unique sequences were generated using the unique.seqs command. A count file containing group information was generated using the count.seqs command. Sequences were aligned to the Silva SSUREF database (v102) using the align.seqs command. Sequences not overlapping in the same region were removed using the screen.seqs command. Sequences were preclustered using the pre.cluster command. Chimeras were removed using UCHIME [31], and any sequences that were not of known bacterial origin were removed using the remove.seqs command. All remaining sequences were classified using the classify.seqs command. All unique sequences with one or two members (single/doubletons) were removed using the AWK command in UNIX. A distance matrix was constructed for the aligned sequences using the dist.seqs command. Sequences were classified at the 97% level with the Ribosomal Database Project (RDP) Naive Bayesian Classifier [32] using a manually constructed training set containing sequences sourced from the greengenes 16S rRNA database (version gg_13_5_99 accessed May 2013), the RDP version 9 training set, and all full-length honeybee-associated gut microbiota on NCBI. Operational taxonomic units (OTUs) were generated using the cluster command. OTUs sharing exact taxonomy were merged using the merge.otu command. Representative sequences for each OTU were generated using the get.oturep command. To further confirm taxonomy, resulting representative sequences were subject to a BLAST query using the NCBI nucleotide database. For Alpha diversity analysis, rarefaction curves describing the number of OTUs observed as a function of sampling effort were generated using the rarefaction.single command. For Beta diversity, similarity calculations of the membership and structure between samples were conducted using the dist.shared and pcoa commands. 

16S abundance statistical analysis: We compared the bacterial abundance with a parametric linear mixed-effects model. Prior to analysis, the 16S gene copy numbers were transformed using log transformation to satisfy assumptions of normality. The abundance was compared between propolis treatments, but also between colonies, in order to quantify the colony effect of the propolis treatment. 

Diversity statistical analysis: We examined the diversity within each propolis treatment, and the relationship between diversity and OTU abundance. We first generated rarefaction curves describing the number of OTUs observed as a function of sampling effort. Next, we calculated the Inverse Simpson diversity index for each treatment condition. To investigate potential relationships between the shifts in abundance and the decrease in diversity, we ran a Spearman’s correlation analysis of the bacterial absolute abundance and the inverse Simpson values. 

Relative abundance statistical analysis: First, we conducted a MANOVA analysis of the centered log-ratio transformed (CLR) based on the relative abundance. To examine the effect of the community size, we multiplied the proportional abundance of OTUs by the group- or species-specific 16S rRNA gene copy number and total bacterial 16S rRNA gene copies determined with qPCR for each sample. 

MANOVA was performed on log-converted relative abundances that account for the structure of the microbiome when comparing particular taxa between treatments. To allow the use of parametric multivariate analyses [33], we converted relative OTU abundances into ratios for all OTUs [34] using the software CoDaPack’s centered log ratio (CLR) transformation [35]. These transformations reflect the ratio abundance of all taxa in the data set. Nearly all of these transformed data sets were normally distributed [34]. A few samples slightly deviated from normal values following transformation. Because our sample size was large (n = 45 for each independent variable), these tests were robust in terms of slight deviations from normality. As an additional measure, we used Pillai’s Trace test statistics, also robust in terms of violations of multivariate normality and homogeneity of covariance. 

The MANOVA analyses were performed on CLR-transformed data, with OTUs 1-16 as dependent variables. False discovery rate (FDR) correction was conducted to account for multiple pairwise comparisons. It should be noted that OTUs found in only a few libraries were removed, resulting in 16 OTUs being used for all downstream statistical analyses.

Absolute abundance statistical analysis: Second, we tested for differences in the absolute abundance of microbial communities using pairwise Wilcoxon rank sum tests followed by FDR correction for multiple comparisons. Unlike the MANOVA, the Wilcoxon rank sum comparisons were conducted based on the calculated absolute cell number for each OTU, and do not account for the overall structure of the microbiome. We used both analyses because it is unknown how the individual bacterial species affect one another; whether they exist as an interacting community network, or as individual species groups that occupy unique, relatively isolated mouthpart micro-niches.

We calculated the absolute cell number of community members by determining the total number of 16S gene copies in each sample via universal “bacti-quant” qPCR, and then assigning a portion of this total to each OTU based on the relative proportions generated from next generation sequencing. To obtain cell number estimates, we then corrected for the average number of 16S operon copies associated with each bacterial taxon according to the rRNA operon copy number database [36] (Supplementary Material Table S2).

An analysis of similarities (ANOSIM) was used to determine if the general similarity between groups was greater than or equal to the similarity within groups. Here, an abundance matrix for the top 94 OTUs was generated in MOTHUR using the cluster command. The matrix was then square root transformed and a Bray–Curtis dissimilarity matrix was generated. The global R and pseudo *p*-value were generated using 999 permutations. Analyses were conducted in Primer-e version 6.4.7, JMP_ v11 (JMP_ 1989–2007) and/or SAS_ v9.4 (2013 SAS).

## 3. Results

Microbiome comparison: Based on an ANOSIM analysis of the top 94 OTUs, the mouthpart microbiomes significantly differed by treatment condition (global test statistic: 0.23, 0.1%).

**Figure 1:**
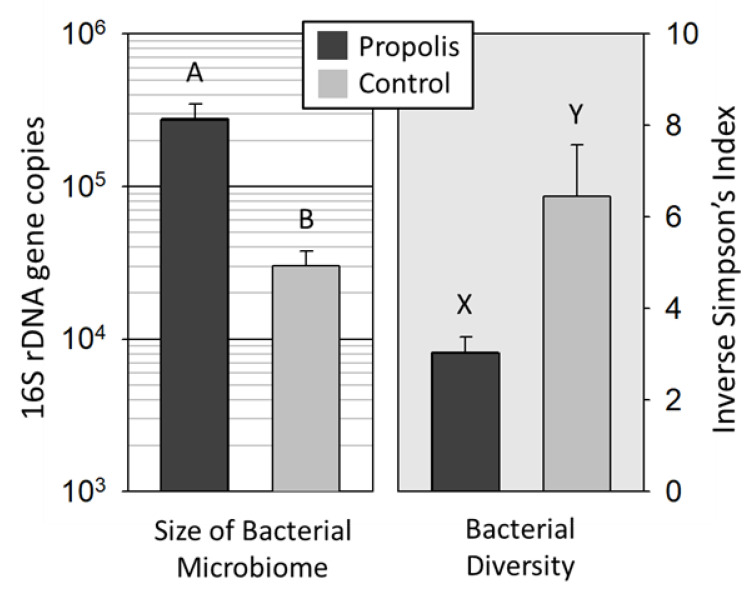
Propolis treatment increased the bacterial abundance but reduced the bacterial diversity. The total bacterial 16S rRNA gene copy number was significantly greater on mouthparts of worker bees in colonies with (A) a propolis envelope compared to the mouthparts of worker bees in colonies without (B) a propolis envelope. Whiskers depict the standard error of the mean. The Inverse Simpson’s Index was significantly lower on the mouthparts of worker bees in colonies with a propolis envelope (X) compared to the mouthparts of worker bees in colonies without a propolis envelope (Y).

16S abundance: The total 16S gene copy number significantly differed between treatments, with the propolis treatment group being significantly greater (F_1,4_ = 27.96, *p* < 0.001) (Figure 1). There was no significant difference between source colonies within the same treatment group (F_5,84_ = 0.5008, *p* = 0.7352). Because colonies within treatments did not differ, downstream analyses did not consider the colony source as a factor. 

Diversity: Next generation sequencing returned 3,875,345 quality trimmed reads (<400 bp) for the 96 amplicon libraries generated, averaging 41,669 reads per library (Supplementary Material Table S3). A total of 1687 OTUs were resolved at 97% similarity. The top 19 OTUs and a 20th group consisting of ‘other’ (Σ OTUs 20-1687) represented 92% and 8% of the total sequences, respectively (Supplementary Material Table S4).

**Figure 2:**
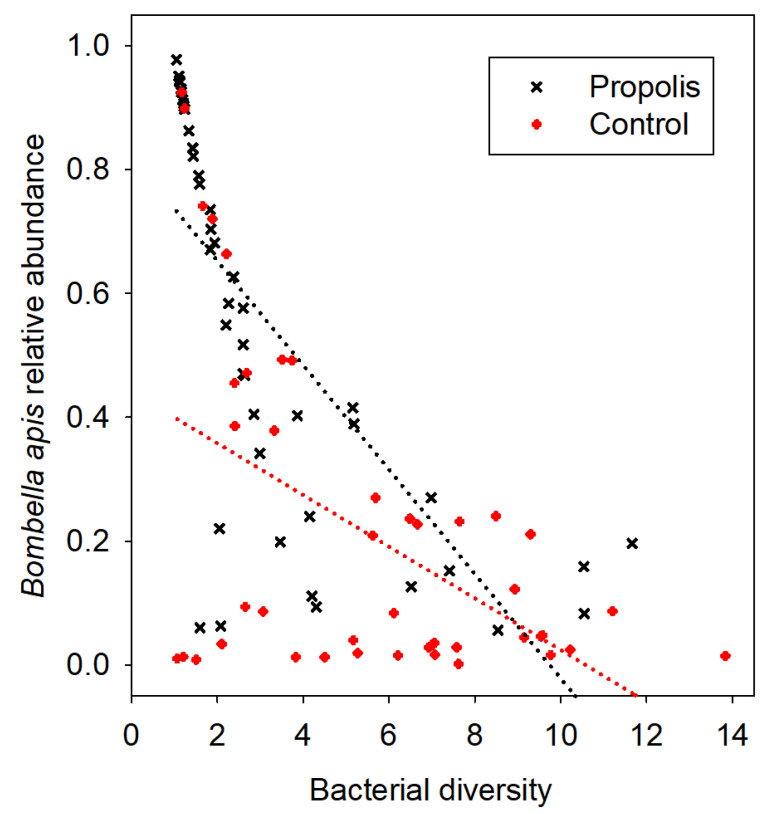
The primary effect of propolis in restructuring the mouthpart microbiome. The *y*-axis shows the variation in the relative abundance of the dominant mouthpart bacterium (*Bombella apis*). The *x*-axis is the total mouthpart diversity (inverse Simpson), where an increased value represents an increased diversity, and the different symbols represent propolis addition or no propolis addition (control). Considering both states, the relationship was significantly negative, but compared to the control, the relationship with added propolis explains significantly greater variation between the dominant bacterium and the diversity of the mouthpart microbiome. Linear regression of the relative abundance of *Bo. apis* with inverse Simpson values; propolis: adjusted R-sq = 0.5, *p* < 0.0001, and control: adjusted R-sq = 0.25 *p* = 0.0003.

We found significantly less bacterial diversity on the mouthparts of workers from propolis-treated colonies compared to the mouthparts of workers from colonies without a propolis envelope (Figure 1) (Z *=* 4.05, *p* < 0.0001). The standard error around the mean diversity was lower in the microbial communities of bees from colonies with propolis compared to the communities of bees from colonies without propolis (Figure 1). The results of the OTU analysis show that *Bombella apis*, *Lactobacillus kunkeei*, and *Fructobacillus fructosus* increased in absolute abundance in the samples collected from colonies treated with propolis. However, the primary effect of propolis was the restructuring of the mouthpart microbiome community by the dominant bacterial species *Bo. apis* (Figure 2). Given the dominance of *Bo. apis* and its correlation with diversity, regardless of the treatment condition (Figure 2), we performed an ANCOVA post-hoc to explore the association of *Bo. apis* with diversity in the context of our independent variable. When the diversity explained by the relative abundance of *Bo. apis* in the mouthpart microbiome was removed from the model, the adjusted means for diversity did not differ by treatment (propolis = 4.3, control = 5.1; F_1, 90_ = 0.4, *p* = 0.53). The interaction term (*Bo. apis* by treatment condition) was not significant (F_1, 89_ = 0.03, *p* = 0.86), meeting the assumption of homogeneous regression slopes required by ANCOVA. 

**Figure 3:**
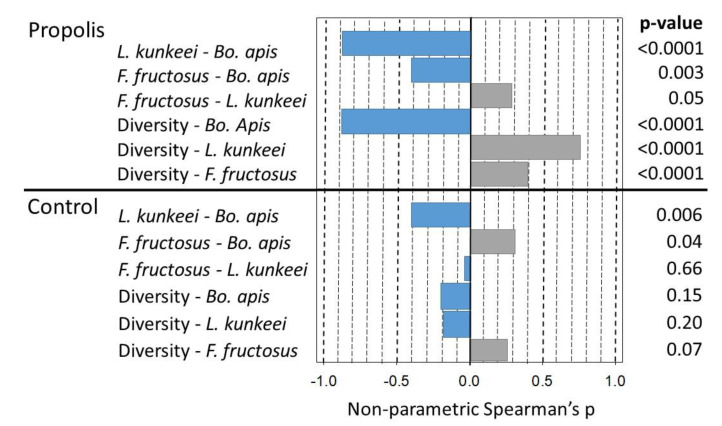
Spearman’s correlations comparing treatments, the absolute abundance of operational taxonomic units (OTUs), and the microbiome diversity. Treatments are propolis and the control (no propolis). The pairwise species correlations depict a positive (gray) or negative (blue) association between the absolute abundance of each species. The mouthpart microbiome diversity is calculated as inverse Simpson’s, which increases with numerical values. Therefore, positive correlations between species and diversity (in gray) depict a greater absolute abundance of species associated with an increased mouthpart diversity. Negative correlations (in blue) depict a lower species absolute abundance associated with an increased mouthpart diversity.

To investigate potential relationships between the shifts in abundance and changes in diversity, we ran a Spearman’s correlation analysis for the bacterial absolute abundance of *Bo. apis*, *L. kunkeei*, and *F. fructosus* and the Inverse Simpson values obtained from the propolis treatment. We found a strong negative relationship between *Bo. apis* and *L. kunkeei* (r_s_ = −0.90, *p* < 0.001) and *Bo. apis* and *F. fructosus* (r_s_ = −0.42, *p* < 0.003). Additionally, while the *Bo. apis* abundance was negatively associated with the mouthpart microbiome diversity (r_s_ = −0.89, *p* < 0.0001), both the *L. kunkeei* and *F. fructosus* abundance associated positively with the mouthpart microbiome diversity (r_s_ = 0.75, *p* < 0.001 and r_s_ = 0.39, *p* < 0.006, respectively (Figure 3)). From the mouthparts of non-propolis colonies, we found no significant correlation between either of the three bacteria and diversity, though we did find a similar negative relationship between *L. kunkeei* and *Bo. apis* and a positive relationship between *Fructobacillus* and *Bo. apis* (r_s_ = −0.41, *p* < 0.006 and r_s_ = −0.31, *p* < 0.04, respectively (Figure 3)). 

Relative abundance: The multivariate analysis (MANOVA) result based on centered log ratios differed by treatment (Pillai’s Trace = 0.44, F = 3.51, df = (75), *p* < 0.0001). Pairwise post-hoc comparisons resulted in ten significant differences following FDR correction for multiple comparisons. *Bo. apis* and *F. fructosus* displayed a greater abundance on mouthparts of samples collected from colonies treated with propolis (F(1, 91) = 49.67, *p* < 0.0001 and F(1, 91) = 29.71, *p* < 0.0001, respectively), whereas *Pseudomonas*, *Pseudoalteromonas*, *Streptococcus*, *Serratia*, *Microbacterium*, *Propionibacterium, Enterobacteriaceae*, and ‘other’ exhibited a lower abundance (F(1, 91) = 37.78, *p* < 0.0003, respectively) (Supplementary Material Table S5).

Absolute abundance: Comparisons between treatments based on Wilcoxon rank sum tests returned three significant differences (Supplementary Material Table S5). In agreement with the MANOVA results, *Bo. apis* and *F. fructosus* displayed a greater abundance on mouthparts of samples collected from colonies treated with propolis (Z = −5.90, *p* < 0.0009 and Z = −5.11, *p* < 0.0009, respectively). Additionally, *L. kunkeei* exhibited a greater absolute abundance on mouthparts of samples collected from colonies treated with propolis (Z = −3.67, *p* < 0.001). 

## 4. Discussion

This study adds to the paradigm of social immunity in honey bees and other social groups [13]. The presence of a propolis envelope within the nest cavity significantly altered the bacterial abundance (Figure 1) and diversity (Figure 1, Figure 2 and Figure 3) on the honey bee mouthparts. In colonies with a propolis envelope, the mouthparts of worker bees had a significantly greater bacterial abundance but significantly lower bacterial diversity, compared to colonies without a propolis envelope, indicating that propolis acts as a selective agent mitigating the microbiome structure and size. Accounting for the majority increase in microbiome size, growth of the dominant mouthpart bacterium significantly increased with the addition of propolis, suggesting that these two factors work in concert to promote a hygienic hive environment.

It is well-established that propolis reduces the expression of individual antimicrobial peptides [3,6], but here, we show an additional way in which propolis may improve colony health, i.e., by altering the colony microbiome, to favor beneficial or commensal bacteria that outcompete potentially pathogenic microbes (Figure 2). Based on the identification of the bacteria and the diversity indices of microbial communities in colonies with or without a propolis envelope (Figure 3), propolis significantly changed the mouthpart microbiome community structure, reducing various potentially pathogenic bacteria and promoting the proliferation of commensal and beneficial bacteria on the honey bee mouthparts. More generally, the taxonomic similarities of microbiomes found on the mouthparts of worker bees from all colonies regardless of treatment suggest a core (native) mouthpart microbiome. The mouthpart microbiome provides a first line of defense against opportunistic and pathogenic bacteria and fungi for bees, and this effect may be enhanced when bees are exposed to a propolis envelope within the nest cavity. 

The major effect of propolis exposure was an increase in both the relative and absolute abundance of *Bo. apis* on the mouthparts of bees (Figure 2). *Bo. apis* is abundant and prevalent on worker mouthparts of bees in general, and so may be considered the dominant core member of the mouthpart microbiome [15,16]. *Bo. apis* is consistently found in environments toxic to most bacteria, such as the aerobic, acidic, and high sugar osmolarity conditions found in food stores (stored pollen and nectar), royal jelly-associated niches (worker hypopharyngeal glands and crop, and larval gut), and queen body niches (mouthparts and throughout the guts) [15,16,17,18,19,21,37,38]. *Bo. apis* is the most abundant bacterium on the queen mouthparts and in the midguts of healthy queens, indicating that this bacterium is non-pathogenic and may provide a protective effect, analogous to *Snodgrasella alvi* in the worker gut [14,16]. *Bo. apis* can produce antifungal metabolites, which were shown to protect developing larvae against fungal infections [39]. Exposure to propolis altered the worker bee mouth microbiome so that it more greatly resembled that of the queen mouthpart microbiome [16], suggesting a beneficial social immune function of *Bo. apis* in a healthy honey bee colony. 

Along with *Bo. apis,* there was a significant increase in the absolute abundance of the fructophilic bacterial species *Lactobacillus kunkeei* and *Fructobacillus fructosus* in the mouthpart microbiome of bees with a propolis envelope. This finding suggests that these three species have co-evolved together in the presence of highly antimicrobial hive components, such as royal jelly, honey, pollen, and propolis [19,20,26]. As well as being putative core mouthpart microbiome species, each of these three bacterial species have demonstrated antimicrobial activity. Feeding *Bo. apis* to worker bees was associated with a statistical decrease in *Nosema* spores—a microsporidian parasite that targets midgut cells [38]. *L. kunkeei* displays antimicrobial activity against the bacterium *Melissococcus plutonius*—a causative pathogen of European foulbrood [40]. *L. kunkeei* and *F. fructosus* both exhibit antimicrobial activity against the bacterium *Paenibacillus larvae*—the causative pathogen of American foulbrood [26]. Coupled with the demonstrated antimicrobial activity of *Bo. Apis, L. kunkeei,* and *F. fructosus*, the addition of propolis to the hive environment could provide additional antimicrobial support to honey bees.

The abundance of *Bo. apis* correlated with a significant decrease in the overall mouthpart microbiome diversity (inverse Simpson values), suggesting that this species either drives the structure of the mouthpart microbiome or is better able to dominate the mouthpart niche because of the antimicrobial properties of propolis, or both. With the presence of a propolis envelope, the negative correlation of *Bo. apis* with *L. kunkeei*, and diversity in general was amplified (Figure 3). When we examined *Bo. apis* as a covariate in an ANCOVA model, the significant difference in diversity between the treatment conditions was lost. The significant negative association of *Bo. apis* with diversity, regardless of the independent variable (Figure 2), indicates that *Bo. apis* is the major microbial factor controlling bacterial diversity. This further supports a potential mechanism of propolis addition; propolis and *Bo. apis* may act synergistically to control bacterial diversity.

Although a few bacteria increased, most species of bacteria significantly decreased in the presence of a propolis envelope, including *Pseudomonas*, *Pseudoalteromonas*, *Streptococcus*, *Serratia*, *Microbacterium*, *Propionibacterium,* and *Enterobacteriaceae*. *Pseudomonas*, *Pseudoalteromonas*, *Streptococcus*, *Microbacterium,* and *Enterobacteriaceae* are all genera that are found in a variety of organisms and environments world-wide and may be transient bacteria brought into the honey bee mouthpart microbiome from the pollination environment. Alternatively, many of these species may have a relatively permanent niche in honey bee colonies. For example, *Serratia marcescens* is a widespread opportunistic pathogen of many plants and animals. This well-known opportunistic bacterium was recently isolated from *Varroa* mites and from the hemolymph of dead and dying honey bees [41]. Genomic analysis revealed that this particular strain of *S. marcescens* was well-equipped for survival in honey bee hives, possessing genes not found in any other strain of *S. marcescens* [42]. This suggests that many bacteria are omnipresent in the hive environment [18,19,20], and further advocates for the generalized antimicrobial activity of propolis against transient or resident opportunistic bacteria in honey bee colonies.

## 5. Conclusions

We found that propolis refined the worker mouthpart microbiome, encouraging the growth of resident (core) commensal or beneficial bacteria and decreasing the relative abundance of putative opportunistic pathogens. The synergistic effect of propolis plus the dominant mouthpart bacterium suggests a variety of hypotheses, both evolutionary and ecological. This result adds to the paradigm of social immunity in honey bees, highlighting mechanisms of disease susceptibility and transmission. Enabling colonies to enhance their own social immunity could be an asset to beekeepers and bee breeders alike. The maintenance of honey bee colonies in man-made hive boxes that do not promote the deposition of a propolis envelope within the nest cavity has occurred only relatively recently in the evolutionary history of managed honey bee colonies. The lack of a propolis envelope within managed colonies could allow for an increase in opportunistic and pathogenic microbes and present a challenge to the health of already stressed colonies. Encouraging colonies to deposit a natural propolis envelope, by using boxes with rough textured or unfinished interior surfaces, would result in multiple health benefits for the colony. 

## Supplementary Materials

The following are available online at https://www.mdpi.com/2075-4450/11/7/453/s1, Table S1: Propolis validation, Table S2: Copy number conversion, Table S3: Read summary, Table S4: Top 20 OTU abundance, Table S5: Statistical summary.
